# Prediction of 30-Day Complicated Clinical Course in Acute Pulmonary Embolism: A Prospective Cohort Comparison of the ESC, Bova, and Modified FAST Models

**DOI:** 10.3390/diagnostics16182991

**Published:** 2026-09-16

**Authors:** Melis Efeoğlu Saçak, Emre Kudu

**Affiliations:** Department of Emergency Medicine, Marmara University School of Medicine, 34899 Istanbul, Türkiye; melisefeoglusacak@gmail.com

**Keywords:** pulmonary embolism, risk stratification, prognosis, Bova score, modified FAST score, emergency medicine

## Abstract

**Background/Objectives:** Risk stratification of hemodynamically stable acute pulmonary embolism guides the intensity of monitoring and the site of care, but the available tools use different variables and may classify the same patients differently. We aimed to compare the prognostic performance of three established models for predicting a complicated clinical course. **Methods:** In a prospective single-center cohort, consecutive hemodynamically stable adults with computed tomography-confirmed acute pulmonary embolism in the emergency department were enrolled. The European Society of Cardiology (ESC) algorithm, the Bova score, and the modified FAST score were subsequently calculated from prospectively collected baseline data. The primary outcome was a complicated clinical course within 30 days (a composite of death, hemodynamic collapse, cardiac arrest, mechanical ventilation, or rescue reperfusion). Discrimination was assessed by the area under the curve (AUC) with DeLong confidence intervals (CI) and pairwise comparison. Prognostic classification performance was assessed at each model’s prespecified intermediate–high-risk threshold. **Results:** Of 383 patients (median age 72 years, 51.7% women), 56 (14.6%) developed a complicated course. Discrimination was modest across the three tools: modified FAST AUC 0.680 (95% CI 0.608–0.752), Bova 0.663 (0.587–0.740), and ESC 0.617 (0.560–0.673). No statistically significant pairwise difference in AUC was detected (all Holm-adjusted *p* > 0.3). Classification characteristics differed substantially: the modified FAST score was the most sensitive (sensitivity 53.6%, specificity 74.0%), whereas Bova Stage III was the most specific (specificity 95.7%, sensitivity 14.3%), with the ESC algorithm intermediate (sensitivity 26.8%, specificity 86.2%). Agreement between the tools for intermediate–high-risk classifications was poor to fair (Cohen’s kappa 0.12–0.41). **Conclusions:** The three tools showed modest discrimination for a 30-day complicated course, and no statistically significant pairwise differences in AUC were detected. They nevertheless classified markedly different proportions of patients as intermediate–high risk and showed distinct sensitivity–specificity profiles. Because the tools carry distinct sensitivity–specificity trade-offs and are not interchangeable, the choice among them should match the clinical question, and no single score should determine treatment alone.

## 1. Introduction

Acute pulmonary embolism remains a common and potentially lethal cardiovascular emergency, with a reported 30-day mortality of roughly 10% in unselected cohorts [1,2]. The clinical spectrum is wide. Patients who present with hemodynamic instability face a very high early mortality and clearly need reperfusion, but they represent only a small fraction of the total. The far larger group arrives hemodynamically stable, and this group is not uniform: it contains patients who will remain stable and others who will deteriorate within days [3].

A subset of stable patients carries a real risk of early adverse events, including death, hemodynamic collapse requiring pharmacologic or mechanical circulatory support, and recurrent embolism [4,5]. Identifying these patients in the emergency department matters because the decision affects where the patient is monitored, whether intensive care is used, and how closely the patient is watched for the deterioration that would trigger rescue reperfusion [6,7]. Guidelines therefore emphasize structured early risk assessment for normotensive acute pulmonary embolism [8].

Several tools have been developed for this task, and they differ in what they measure. The European Society of Cardiology algorithm defines an intermediate–high-risk group by the combination of a positive simplified Pulmonary Embolism Severity Index [9,10], right ventricular dysfunction, and troponin elevation [11]. The Bova score assigns points to systolic blood pressure, heart rate, troponin, and right ventricular dysfunction and sorts patients into three stages [4]. The modified FAST score combines troponin, syncope, and tachycardia and can be computed quickly at the bedside [12,13]. Because the tools use different variables, they can place the same patient in different risk categories, and it is not obvious which performs best for predicting a clinically meaningful complicated course, which combines death with the early deteriorations that drive escalation of care [14].

Comparative data on the performance of these scores are limited and inconsistent in the literature. In one prospective cohort, the modified FAST score discriminated best and the ESC algorithm least [13], whereas a later registry analysis found the ESC algorithm on top and the Bova score lowest [15]. More recently, Mirambeaux et al. directly compared the ESC, Bova, modified FAST, and PEITHO-3 models for predicting a 30-day complicated course. The models identified substantially different proportions of patients as intermediate–high risk. The Bova score classified the smallest proportion of patients in this category but showed the highest specificity, whereas the modified FAST score classified a broader group as intermediate–high risk [16]. These previous studies establish that the models can yield substantially different risk classifications, but their comparative findings have not been consistent, and several analyses were performed post hoc using earlier registry or cohort data. The present study was therefore designed to prospectively compare the ESC algorithm, Bova score, and modified FAST score in a contemporary cohort of consecutive patients presenting to the emergency department and to assess whether previously reported differences between these models were reproducible.

## 2. Materials and Methods

### 2.1. Study Design and Setting

This was a prospective observational cohort study carried out in the adult emergency department of a tertiary-care academic hospital between April 2025 and April 2026. Consecutive patients with confirmed acute pulmonary embolism were assessed for eligibility during the enrollment period. The study was approved by the Marmara University Clinical Research Ethics Committee (approval no. 09.2024.446, approval Date: 22 April 2024), and written informed consent was obtained from every participant.

### 2.2. Study Population

Adults aged 18 years or older were eligible when acute pulmonary embolism was confirmed by computed tomographic pulmonary angiography, and the patient was hemodynamically stable at presentation. Hemodynamic stability was defined as a systolic blood pressure of at least 90 mmHg without vasopressor support and without signs of shock. Patients who underwent reperfusion at the time of diagnosis were not included. Patients were excluded for pregnancy, a life expectancy shorter than one month, refusal of consent, or inaccessibility for follow-up.

### 2.3. Data Collection and Definitions

Demographic data, comorbidities, presenting symptoms, vital signs, arterial blood gas values, laboratory results, and imaging findings were prospectively collected during the initial emergency department evaluation and subsequent diagnostic workup before any clinical deterioration occurred.

High-sensitivity cardiac troponin I (hs-cTn I) was measured using the Access hs cTn I assay (Beckman Coulter, Brea, CA, USA), with results reported in ng/L. Troponin elevation was defined using the sex-specific 99th percentile upper reference limits applied by our laboratory, 11.6 ng/L for women and 19.8 ng/L for men. Blood sampling was performed during the initial emergency department evaluation. When serial troponin measurements were available, the first value obtained was used for calculation.

Pulmonary Embolism Diagnosis: Acute pulmonary embolism was diagnosed by an intraluminal filling defect on computed tomographic pulmonary angiography, interpreted in the routine radiology workflow.

Right Ventricular Dysfunction: Right ventricular dysfunction was assessed using both computed tomography pulmonary angiography (CTPA) and transthoracic echocardiography in all patients. A positive finding on either modality was sufficient for classification as right ventricular dysfunction. On CTPA, right ventricular enlargement was defined as a right-to-left ventricular short-axis diameter ratio of 1 or greater [17]. Cardiologists performed and interpreted transthoracic echocardiography. Echocardiographic right ventricular dysfunction was defined by the presence of at least two of the following findings: right ventricular dilatation, defined as an end diastolic right ventricular diameter greater than 30 mm in the parasternal view or a right ventricle larger than the left ventricle in the apical or subcostal view; right ventricular free wall hypokinesis; and an estimated systolic pulmonary artery pressure greater than 30 mmHg [4].

The timing of echocardiography relative to CTPA was determined using the examination timestamps recorded in the electronic hospital system. The time from CTPA to echocardiography was calculated in hours. When echocardiography had been performed before CTPA, the interval was recorded as 0 h, indicating no post-CTPA delay.

### 2.4. Risk Stratification Models

For calculation of the ESC, Bova, and modified FAST models, heart rate, systolic blood pressure, and peripheral oxygen saturation were defined as the first values recorded at presentation to the emergency department. These values were used consistently for all three models rather than measurements obtained when pulmonary embolism was subsequently confirmed. The three models were subsequently calculated by the study investigators from these prospectively recorded baseline data for the purposes of the study. These scores were not routinely incorporated into the clinical workflow at our institution, and their calculation was not mandated by the study protocol or used as a predefined basis for treatment or disposition decisions.

#### 2.4.1. ESC Algorithm

The simplified Pulmonary Embolism Severity Index (sPESI) was calculated at diagnosis using six variables: age >80 years, history of cancer, chronic cardiopulmonary disease, heart rate ≥110/min, systolic blood pressure <100 mmHg, and arterial oxygen saturation <90%, with 1 point assigned to each variable. An sPESI score of 0 indicated low clinical risk, whereas a score ≥1 was considered positive [10]. According to the ESC risk-stratification algorithm [11], hemodynamically stable patients with a positive sPESI were classified as intermediate–high risk when both troponin elevation and right ventricular dysfunction were present. Patients with a positive sPESI who did not meet both of these criteria were classified as intermediate–low risk. Patients with an sPESI of 0 were classified as low risk when neither right ventricular dysfunction nor troponin elevation was present; those with an sPESI of 0 but evidence of right ventricular dysfunction and/or troponin elevation were classified as intermediate–low risk.

#### 2.4.2. Bova Score

The Bova score assigns points for systolic blood pressure of 90 to 100 mmHg (2 points), troponin elevation (2 points), right ventricular dysfunction (2 points), and heart rate of 110/min or higher (1 point). Stage I corresponds to 0 to 2 points, Stage II to 3 to 4 points, and Stage III to more than 4 points. Stage III was taken as the intermediate–high-risk category in the comparative analysis [4].

#### 2.4.3. Modified FAST Score

The modified FAST score combines troponin elevation (1.5 points), syncope (1.5 points), and a heart rate of at least 100/min (2 points). A total score of 3 or higher was considered the intermediate–high-risk category for the comparative analysis [13].

### 2.5. Outcome Measures

The primary outcome was a complicated clinical course within 30 days after the diagnosis of pulmonary embolism. A complicated course was a composite of all-cause death, hemodynamic collapse, cardiac arrest, rescue reperfusion therapy, or invasive mechanical ventilation. Hemodynamic collapse was defined, consistent with the original Bova study [4], as a systolic blood pressure below 90 mmHg for more than 15 min or the need for catecholamine administration because of persistent arterial hypotension or shock. Rescue reperfusion referred to systemic thrombolytic therapy administered after the initial diagnosis because of subsequent clinical deterioration. The individual components were not mutually exclusive. When more than one component occurred in the same patient, we recorded each event separately, but counted the patient only once for the primary composite outcome. Secondary outcomes were five-day mortality, intensive care unit admission, and the individual components of the composite.

Thirty-day outcomes were ascertained using hospital records and telephone follow-up. Patients discharged directly from the hospital were contacted during the 30-day follow-up period to determine survival status, subsequent hospitalization, and occurrence of the prespecified outcome events. Thirty-day outcome ascertainment was complete for all 383 patients included in the final analysis.

### 2.6. Sample Size

The target sample size was determined a priori with reference to the prognostic performance reported in a previous comparative validation study [15]. Based on these reported estimates, an anticipated primary outcome rate of 14%, a two-sided alpha level of 0.05, and 80% statistical power, the planned enrollment was set at 360 patients. The calculation was performed using G*Power version 3.1.9.7 [18].

### 2.7. Statistical Analysis

Analyses were performed in R version 4.5.3 (R Foundation for Statistical Computing, Vienna, Austria) and IBM SPSS Statistics for Windows, version 23.0 (IBM Corp., Armonk, NY, USA). Descriptive statistics and baseline group comparisons were performed in SPSS. ROC analyses, DeLong confidence intervals and pairwise comparisons of correlated ROC curves were performed in R using the pROC package (version 1.19.0). The three pairwise *p* values were adjusted using the Holm method with the base R p.adjust function. Wilson confidence intervals for proportions were calculated using the DescTools package (version 0.99.60), and Cohen’s kappa was calculated in R using the irr package.

Continuous variables were presented as median and interquartile range (IQR), since the distributions departed from normality on the Shapiro–Wilk test, and were compared between patients with and without the primary outcome using the Mann–Whitney U test. Categorical variables were summarized as counts with percentages and compared with the chi-square test, or the Fisher exact test.

Discrimination for the primary outcome was quantified by the area under the receiver operating characteristic curve, with 95% confidence intervals obtained by the DeLong method. The continuous score was used for the Bova and modified FAST models. For the ESC algorithm, risk categories were treated as an ordinal variable and coded as 0 for low risk, 1 for intermediate–low risk, and 2 for intermediate–high risk. The three pairwise differences between AUCs were tested using the DeLong test for correlated curves, and the resulting *p* values were adjusted using the Holm method. At each model’s intermediate–high-risk threshold, sensitivity, specificity, positive and negative predictive values, and positive and negative likelihood ratios were calculated against the primary outcome. Wilson 95% confidence intervals were used for sensitivity, specificity, PPV, and NPV, whereas 95% confidence intervals for LR+ and LR− were calculated using the logarithmic method. Agreement between the models’ binary intermediate–high-risk classifications was measured with Cohen’s kappa. All tests were two-sided with an alpha of 0.05. Complete data were available for all variables required to calculate the ESC, Bova, and modified FAST models in all 383 patients included in the analysis. No imputation of missing predictor data was therefore required.

## 3. Results

### 3.1. Study Population

**Figure 1:**
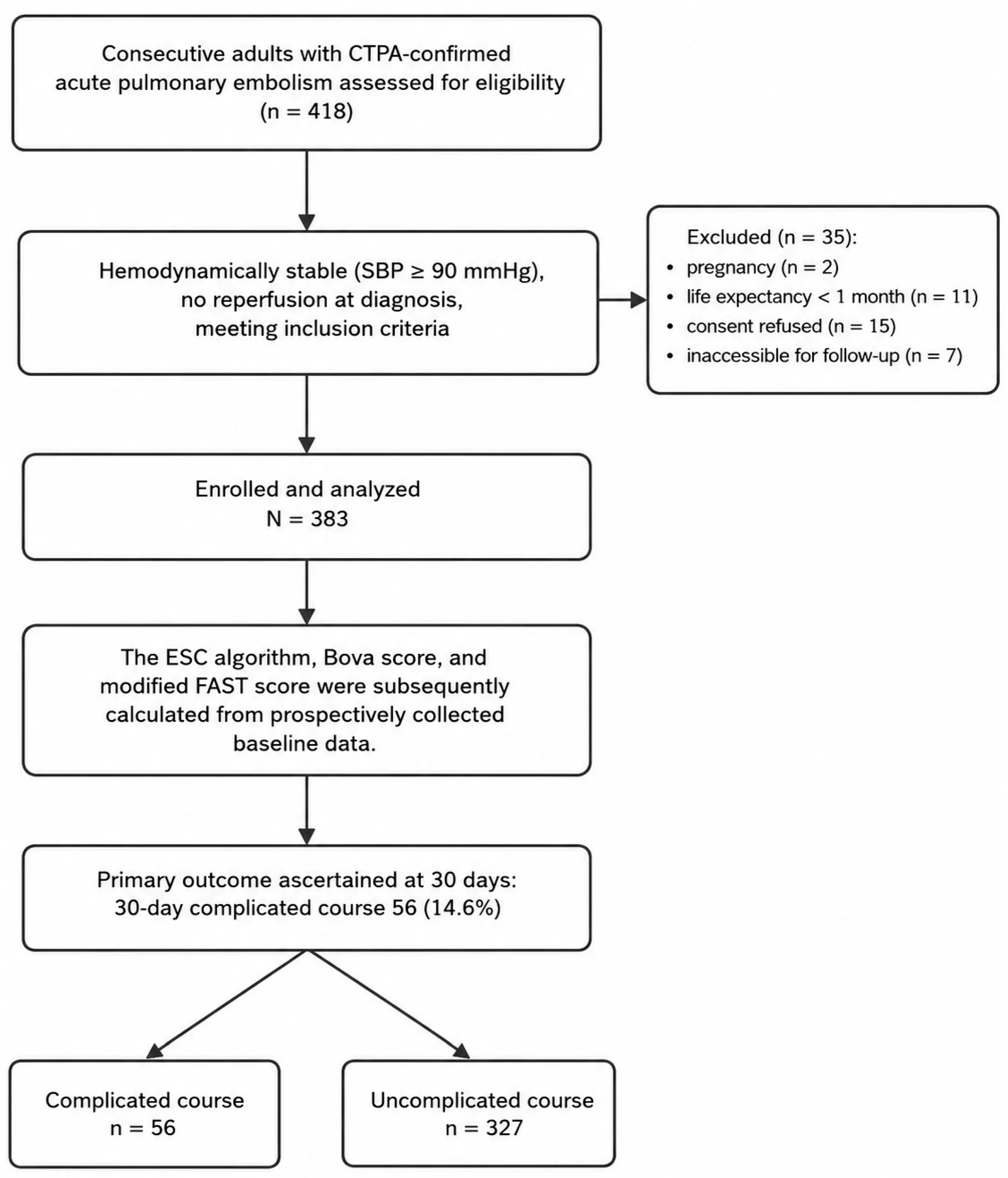
Patient flow. Of 418 patients assessed for eligibility, 35 were excluded, leaving 383 hemodynamically stable adults with CT-confirmed acute pulmonary embolism. The risk models were subsequently calculated from prospectively collected baseline data, and 30-day outcome ascertainment was complete for all patients (*n* = 383).

During the enrollment period, 418 patients with CTPA-confirmed acute pulmonary embolism were assessed for eligibility. Of these, 35 patients were excluded: 2 because of pregnancy, 11 because of a life expectancy shorter than one month, 15 because they declined to provide informed consent, and 7 because they were inaccessible for follow-up. The remaining 383 hemodynamically stable patients were included in the final analysis (Figure 1).

The median (IQR) age was 72 (62.5–81) years, and 198 patients (51.7%) were women. Troponin was elevated in 220 patients (57.4%). All 383 patients underwent both CTPA and transthoracic echocardiography, and right ventricular dysfunction was present in 75 patients (19.6%) by any modality. The median recorded time from CTPA to echocardiography was 1 h (IQR 0 to 1). Ninety-one patients were discharged from the emergency department, 228 were admitted to a ward, and 64 were admitted to an intensive care unit. The median (IQR) length of stay was 5 (2–9) days.

### 3.2. Baseline Characteristics

A complicated 30-day course developed in 56 patients, so baseline features were compared between these patients and the 327 without the outcome (Table 1). Patients who developed a complicated course had a higher heart rate (median 110 versus 91/min, *p* < 0.001) and a lower oxygen saturation (88% versus 94%, *p* < 0.001). Dyspnea was more common in this group (83.9% versus 68.8%, *p* = 0.032), as were troponin positivity (71.4% versus 55.0%, *p* = 0.032), right ventricular dysfunction by any modality (35.7% versus 16.8%, *p* = 0.002), and a CTPA right-to-left ventricular ratio of 1 or greater (19.6% versus 7.0%, *p* = 0.005). Age and sex did not differ between the groups.

### 3.3. Clinical Outcomes

The primary outcome, a complicated clinical course within 30 days, occurred in 56 of 383 patients (14.6%). Its components were invasive mechanical ventilation in 40 patients, 30-day all-cause death in 27, cardiac arrest in 27, hemodynamic collapse in 33, and rescue thrombolysis in 9. Because individual patients could experience more than one component, these counts exceeded the number of patients with the composite outcome. Five-day mortality was rare, with three deaths, and no patient died in the emergency department (Table 2).

### 3.4. Risk Classification

**Figure 2:**
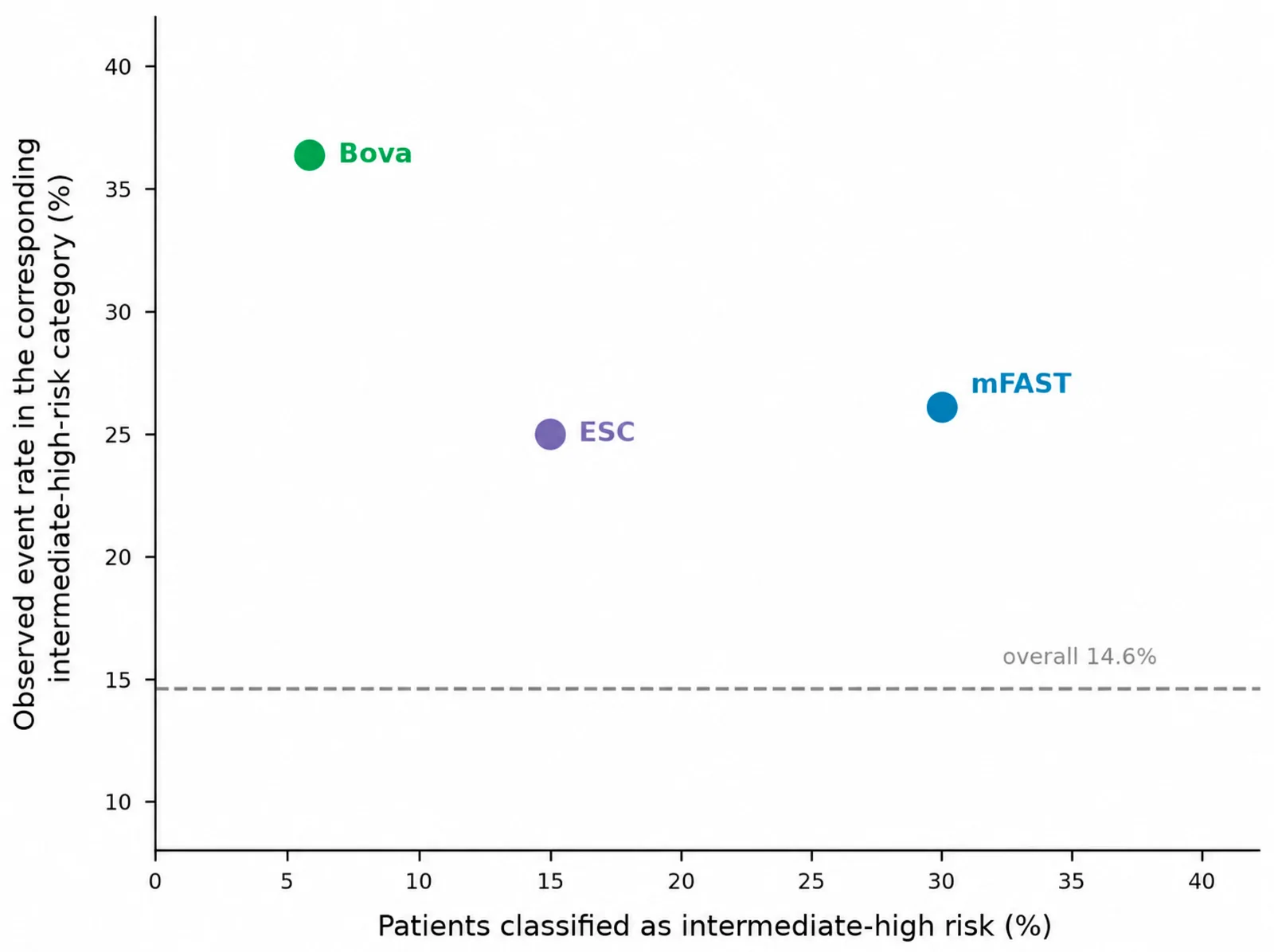
Trade-off between the proportion of patients classified intermediate–high risk (x-axis) and the observed event rate within the intermediate–high-risk class (y-axis). The dashed line marks the overall event rate (14.6%).

The three models placed different proportions of the cohort in the intermediate–high-risk category. The modified FAST score classified 115 of 383 patients (30.0%) as intermediate–high risk, the ESC algorithm classified 60 (15.7%), and Bova Stage III classified only 22 (5.7%) (Figure 2, Table 3). These categories are model specific and do not represent identical risk definitions.

### 3.5. Prognostic Performance

**Figure 3:**
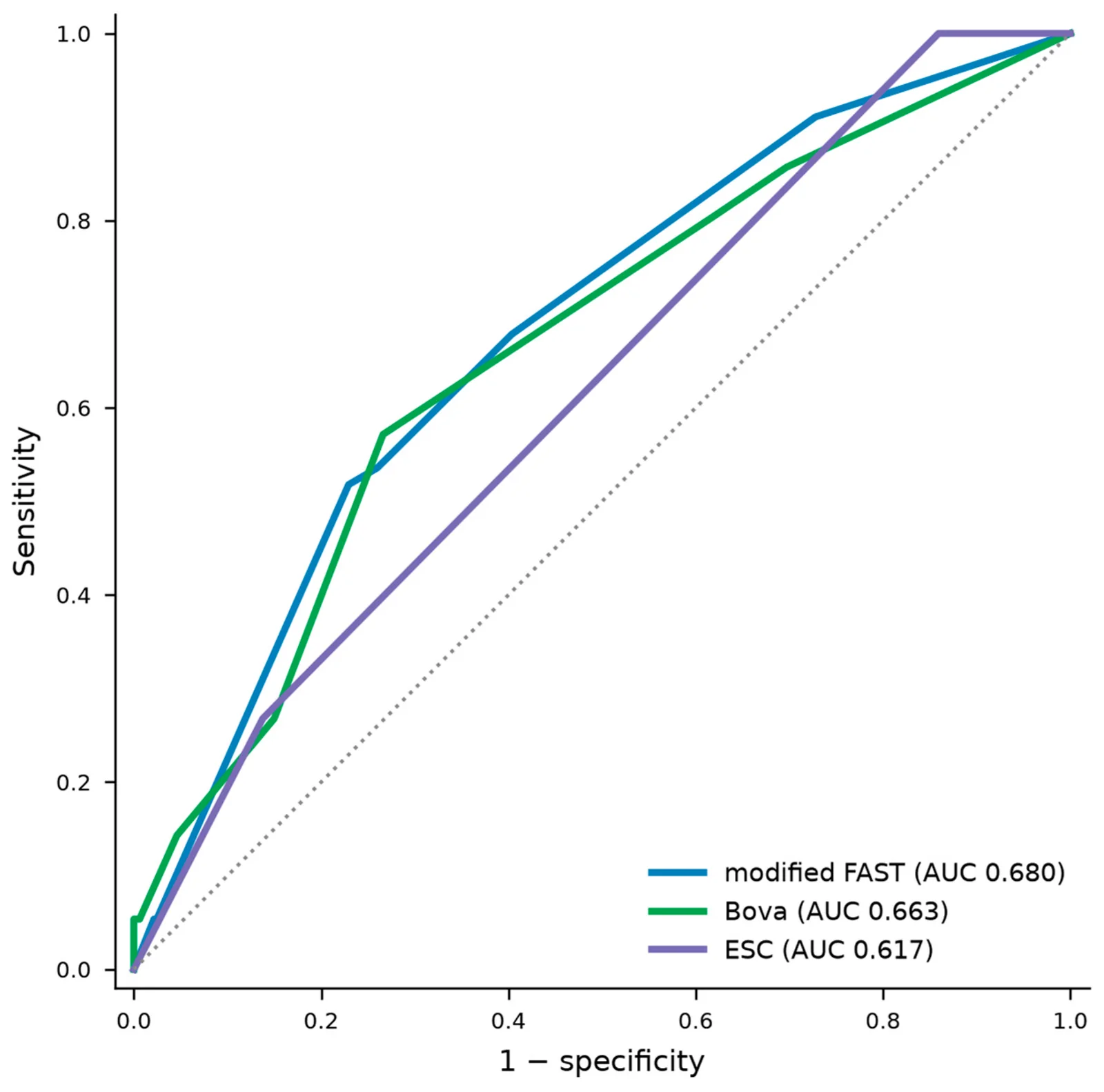
Receiver operating characteristic curves for the three risk models predicting a 30-day complicated course. Areas under the curve: modified FAST 0.680, Bova 0.663, ESC 0.617.

Discrimination for the primary outcome was modest across the three models. The modified FAST score had the highest area under the curve, at 0.680 (95% confidence interval 0.608–0.752), followed by the Bova score at 0.663 (0.587–0.740) and the ESC algorithm at 0.617 (0.560–0.673) (Figure 3, Table 4). None of the three pairwise differences reached statistical significance: modified FAST versus ESC (difference 0.063, DeLong *p* = 0.110; Holm-adjusted *p* = 0.329), Bova versus ESC (difference 0.046, *p* = 0.200; adjusted *p* = 0.401), and modified FAST versus Bova (difference 0.017, *p* = 0.693; adjusted *p* = 0.693).

**Figure 4:**
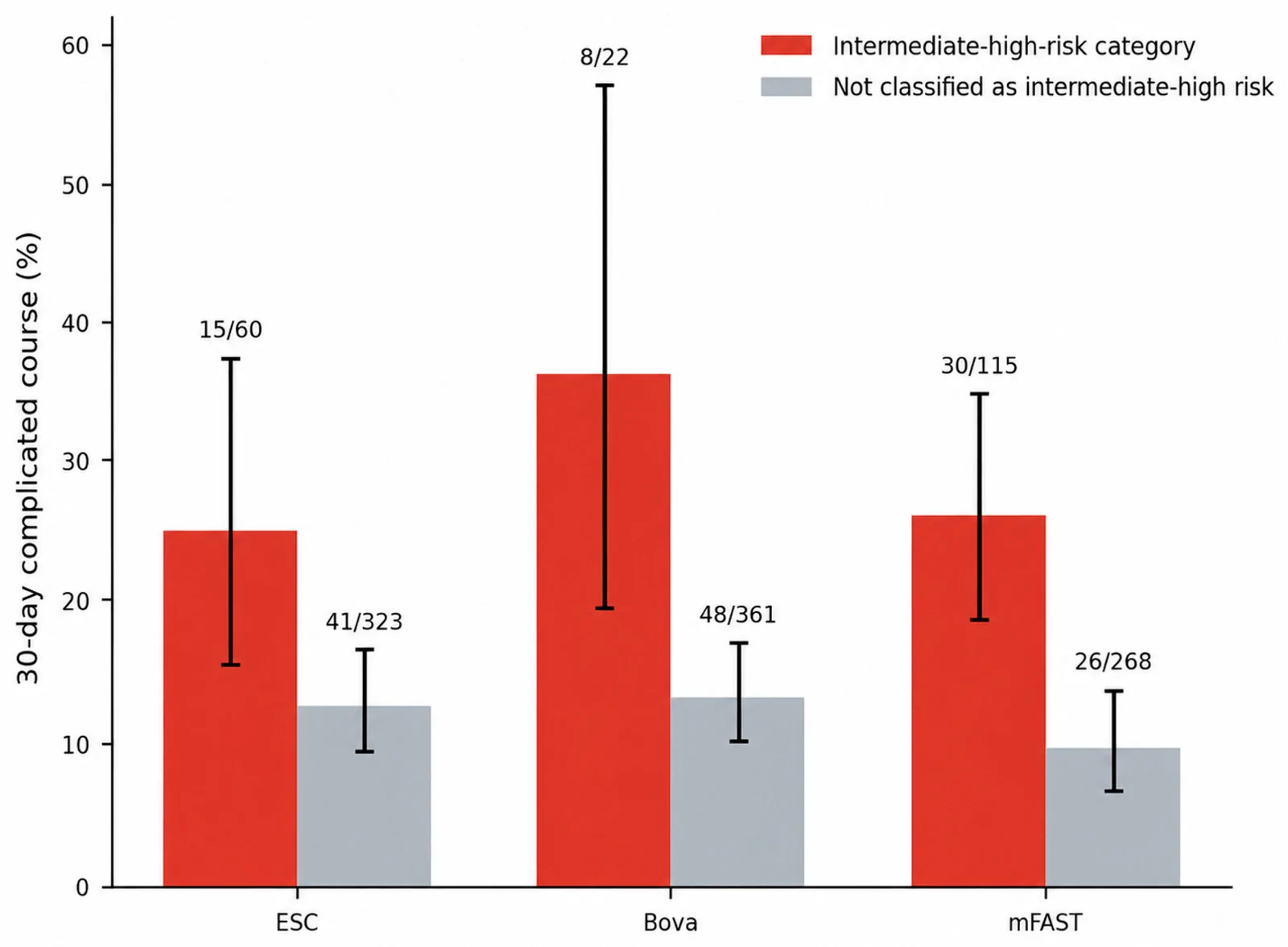
Observed 30-day complicated-course rate in the intermediate–high-risk versus not-intermediate–high-risk class for each model, with Wilson 95% confidence intervals. Counts above bars are events/n.

Classification performance at the prespecified intermediate–high-risk thresholds differed (Table 4). The modified FAST score gave the highest sensitivity, at 53.6% (95% confidence interval 40.7–66.0), with a specificity of 74.0% (69.0–78.5). The ESC algorithm had a sensitivity of 26.8% (17.0–39.6) and a specificity of 86.2% (82.1–89.6). Bova Stage III was the most specific, at 95.7% (92.9–97.4), but identified only a small intermediate–high-risk minority, with a sensitivity of 14.3% (7.4–25.7). Negative predictive values ranged from 86.7% to 90.3% and were influenced by the cohort event rate. The observed event rate among patients classified as intermediate–high-risk was 26.1% for the modified FAST score, 25.0% for the ESC algorithm, and 36.4% for Bova Stage III, compared with 9.7%, 12.7%, and 13.3% among those not classified intermediate–high-risk (Figure 4).

### 3.6. Agreement Between Models

Agreement between the models’ binary classifications was poor to fair (Table 5). Cohen’s kappa was highest between the ESC algorithm and the Bova score at 0.414 (0.279–0.549), intermediate between the Bova and modified FAST scores at 0.216 (0.130–0.303), and lowest between the ESC algorithm and the modified FAST score at 0.115 (0.016–0.214). The three tools therefore identified substantially different sets of patients as intermediate–high-risk.

## 4. Discussion

In this prospective cohort of 383 hemodynamically stable patients with acute pulmonary embolism, a complicated clinical course within 30 days occurred in 14.6%. The three risk-stratification tools showed modest discrimination for this outcome. The modified FAST score had the numerically highest area under the curve (0.680), followed by the Bova score (0.663) and the ESC algorithm (0.617), but no statistically significant pairwise AUC difference was detected. The models differed more clearly in their classification characteristics than in their overall discrimination. They classified substantially different proportions of patients as intermediate–high risk and showed poor to fair agreement at their prespecified thresholds.

### 4.1. Comparison with Previous Studies

The discrimination we observed is consistent with the published performance of these tools, which is itself heterogeneous. A single-center prospective cohort reported high areas under the curve for the modified FAST score and the Bova score, at 0.82 and 0.80 for a 30-day adverse outcome [13], whereas a large registry validation reported much lower values of 0.603 for the modified FAST score and 0.586 for the Bova score against 30-day mortality [19]. Differences in study population, predictor measurement, and outcome definition likely contribute to this variability. Another registry-based comparison reported AUCs of 0.68 for the ESC algorithm, 0.67 for the modified FAST score, and 0.64 for the Bova score [15]. These values are close to those observed in our cohort. Cohort studies using all-cause mortality as the endpoint have likewise reported concordance indices in the 0.7 to 0.8 range for these guideline-based scores [20,21,22]. Across these studies, no single model has consistently shown superior discrimination. Our estimates fall within the range reported in previous cohorts and support the same conclusion.

The pattern of risk classification in our cohort is also consistent with the findings of Mirambeaux et al. [16]. In that study, the Bova score classified the smallest proportion of patients as intermediate–high risk and showed the highest specificity for a 30-day complicated course. The modified FAST score identified a substantially larger intermediate–high-risk group. We observed the same general pattern. Bova Stage III classified only 5.7% of our cohort as intermediate–high risk, whereas the modified FAST score classified 30.0%.

Classification characteristics at the prespecified thresholds provide information that is not captured by AUC alone. The modified FAST score, by flagging the largest group, achieved the highest sensitivity, while Bova Stage III, by flagging the smallest, was the most specific. This reflects important differences in how the models define risk. Risk assessment in pulmonary embolism may also be influenced by broader coagulation profiles that are not captured by these scores. Recent prospective data in patients with pulmonary tuberculosis showed that lower protein C and free protein S levels were associated with pulmonary embolism, supporting the potential relevance of hypercoagulability related biomarkers beyond conventional cardiac and imaging markers [23]. Troponin elevation and right ventricular dysfunction are established markers of adverse outcome in acute pulmonary embolism and are incorporated differently across the evaluated models [24]. The Bova score was developed to concentrate risk within a relatively small Stage III group [5,25], and a meta-analysis reported a higher positive predictive value for Bova Stage III than for the ESC intermediate–high-risk category [26]. The ESC algorithm requires the simultaneous presence of a positive sPESI, troponin elevation, and right ventricular dysfunction for classification as intermediate–high risk. In our cohort, its operating characteristics therefore fell between the broader modified FAST classification and the more selective Bova Stage III classification [14]. This ordering of operating characteristics was also observed in our cohort, although no statistically significant pairwise differences in AUC were detected.

### 4.2. Clinical Interpretation

The models produced different intermediate–high-risk classification rates and operating characteristics. Modified FAST had the highest sensitivity, but 26 of the 56 patients with a complicated course were not classified as intermediate–high risk; therefore, its negative predictive value of approximately 90% does not support use as a stand-alone rule-out tool. Bova Stage III had high specificity but identified only 8 of the 56 patients with the outcome, and its positive predictive value was 36.4%. The ESC algorithm showed intermediate operating characteristics. These findings do not establish one model as preferable in all patients. Rather, they show that each model emphasizes a different part of the sensitivity and specificity trade-off. The poor agreement between the tools reinforces that they are not interchangeable, and that a patient’s risk label depends heavily on which tool is applied.

These differences may have practical implications for how risk models are used in different clinical settings [27]. The preferred strategy may depend on the purpose of risk stratification, the availability of diagnostic resources, and the capacity for monitored or intensive care. In settings where monitored care is readily available, a more sensitive strategy may be acceptable because it identifies a broader group of patients who could deteriorate. Where monitored beds or critical care resources are limited, a more specific strategy may help concentrate surveillance on patients with a higher observed event rate.

Feasibility also differs between the models. The modified FAST score does not require assessment of right ventricular dysfunction [13]. The ESC algorithm and Bova score require imaging information in addition to cardiac biomarkers [4,11]. The choice of model may therefore depend not only on prognostic performance, but also on the clinical objective and available resources [28]. When the priority is to minimize missed deterioration and monitored care is readily available, a more sensitive strategy such as the modified FAST score may be preferable because it identifies a broader group of patients at potential risk. In contrast, when monitored or critical care capacity is limited, the higher specificity of Bova Stage III may help prioritize a smaller subgroup with a higher observed event rate [4]. The resources required for score calculation should also be considered, because limited access to echocardiography or other assessment of right ventricular dysfunction may favor the simpler modified FAST approach [15]. The consequences of false-positive and false-negative classifications should therefore be considered alongside feasibility. These resource-based applications were not directly evaluated in our study and should therefore be considered hypothesis-generating rather than practice recommendations, requiring prospective evaluation before they can be used to guide institutional protocols.

The clinical interpretation of the composite outcome also requires some caution. Some components, particularly rescue reperfusion and mechanical ventilation, are influenced not only by disease progression but also by treatment decisions [29]. These decisions may themselves be informed by clinical, biomarker, and imaging findings that overlap with the variables included in the evaluated risk models [30]. Therefore, the association between risk classification and the composite outcome may partly reflect subsequent management decisions in addition to the underlying risk of deterioration.

These scores are not treatment rules. Reperfusion decisions still require clinical evidence of deterioration. They are tools to structure early triage, to inform the level of monitoring, and to support the decision about the site of care, and they are best interpreted alongside the clinical condition and the trajectory of the patient over the first hours and days [31].

### 4.3. Strengths and Limitations

The strengths of this study include its prospective design, consecutive enrollment, and head-to-head comparison of three risk-stratification tools in the same cohort using an identical outcome definition.

Several limitations should be noted. This was a single-center study, and the case mix and the observed event rate may not generalize to other settings. The primary outcome was a composite that combined events of differing severity, from invasive mechanical ventilation to death, which is standard in this field but means that the discrimination reflects a heterogeneous endpoint. Invasive mechanical ventilation accounted for a substantial proportion of the composite and may represent an early supportive intervention during clinical deterioration, frequently overlapping with subsequent events such as hemodynamic collapse, cardiac arrest, or death. Although the composite components were selected in accordance with endpoints used in previous prognostic studies of acute pulmonary embolism, the limited number of remaining events precluded a reliable sensitivity analysis using a narrower endpoint restricted to the most severe PE-related outcomes. This should be considered when interpreting the comparative prognostic performance of the three models. The timing of echocardiography was not standardized. Although the median recorded time from CTPA to echocardiography was only 1 h, short-term changes in right ventricular function may still have affected imaging-based risk classification. Some components of the primary outcome, particularly rescue reperfusion and mechanical ventilation, may also be influenced by clinical decision making and local practice. Although calculation of the ESC, Bova, and modified FAST scores was not mandated as part of routine clinical management, these models are publicly available and are based on routinely accessible clinical, laboratory, and imaging variables. Independent calculation or consideration of the scores by treating physicians could therefore not be excluded. Moreover, individual variables included in the models may themselves have influenced subsequent treatment decisions. These factors introduce potential management-related bias in the association between risk classification and subsequent clinical outcomes. No statistically significant pairwise differences in AUC were detected; however, this finding should not be interpreted as evidence of equivalence between the three models. Differences in threshold-based operating characteristics should therefore be interpreted as comparative descriptions of the models rather than evidence that one should replace another.

### 4.4. Future Directions

The modest discrimination of all three models and the absence of statistically significant pairwise differences in AUC support further prospective evaluation of their threshold characteristics. Multicenter studies with standardized imaging and a common outcome definition would allow the thresholds of each tool to be calibrated to a specific clinical decision, such as eligibility for early discharge or the need for monitored admission. Combining a sensitive tool with a specific one, rather than relying on a single score, is a strategy that merits prospective testing.

## 5. Conclusions

In this single-center prospective cohort of hemodynamically stable patients with acute pulmonary embolism, the ESC algorithm, Bova score, and modified FAST score showed modest discrimination for a heterogeneous 30-day composite outcome. No statistically significant pairwise AUC difference was detected, although the models classified substantially different proportions of patients as intermediate–high-risk and had different operating characteristics. None of the evaluated thresholds demonstrated sufficient performance to exclude deterioration or independently guide treatment. Multicenter validation using standardized predictor assessment, blinded outcome adjudication, and clinically coherent pulmonary embolism-related outcomes is required.

## Figures and Tables

**Table 1:** Baseline characteristics by 30-day clinical course.

Characteristic	Uncomplicated (*n* = 327)	Complicated (*n* = 56)	*p* Value
Age (years)	73 (61–81)	72 (64–79)	0.892
Female sex	176 (53.8%)	22 (39.3%)	0.062
SBP (mmHg)	127 (114–145)	129 (111–150)	0.746
Heart rate (/min)	91 (80–108)	110 (95–124)	<0.001
SpO_2_ (%)	94 (89–97)	88 (83–94)	<0.001
Hypertension	185 (56.6%)	26 (46.4%)	0.206
Diabetes	99/327 (30.3%)	20 (35.7%)	0.512
Active cancer	57 (17.4%)	13 (23.2%)	0.397
Heart failure	38 (11.6%)	8 (14.3%)	0.731
CAD	92 (28.1%)	15 (26.8%)	0.963
CKD	12 (3.7%)	1 (1.8%)	0.702
Concurrent DVT	20 (6.1%)	1 (1.8%)	0.336
Syncope	28 (8.6%)	6 (10.7%)	0.611
Dyspnea	225 (68.8%)	47 (83.9%)	0.032
Chest pain	73 (22.3%)	9 (16.1%)	0.380
Troponin positive	180 (55.0%)	40 (71.4%)	0.032
RV dysfunction (any)	55 (16.8%)	20 (35.7%)	0.002
Echo RV dysfunction	51 (15.6%)	15 (26.8%)	0.063
CT RV/LV ≥ 1	23 (7.0%)	11 (19.6%)	0.005

Values are median (IQR) or n/N (%). *p* values from Mann–Whitney U (continuous) or χ^2^/Fisher exact (categorical). SBP: Systolic blood pressure, SpO_2_: Peripheral oxygen saturation, CAD: Coronary artery disease, CKD: Chronic kidney disease, DVT: Deep vein thrombosis, RV: Right ventricle, CT: Computed tomography, LV: Left ventricle.

**Table 2:** Clinical outcomes.

Outcome	n	% of 383
30-day complicated course (primary)	56	14.6
Invasive Mechanical ventilation	40	10.4
30-day all-cause death	27	7.0
Cardiac arrest	27	7.0
Hemodynamic collapse	33	8.6
Rescue thrombolysis	9	2.3
5-day mortality	3	0.8
ICU admission	64	16.7
Emergency department mortality	0	0.0

Some patients have more than one outcome. ICU: Intensive care unit.

**Table 3:** Risk classification by each model.

Model	Not Classified as Intermediate–High Risk, n (%)	Intermediate-High Risk According to the Model, n (%)	Definition
ESC	323 (84.3)	60 (15.7)	sPESI+ & troponin+ & RV dysfunction
Bova	361 (94.3)	22 (5.7)	Stage III (>4 points)
modified FAST	268 (70.0)	115 (30.0)	Score ≥ 3

sPESI: simplified Pulmonary Embolism Severity Index.

**Table 4:** Discrimination and classification performance for the 30-day complicated course.

Model	AUC (95% CI)	Sensitivity % (95% CI)	Specificity % (95% CI)	PPV % (95% CI)	NPV % (95% CI)	LR+	LR−
ESC	0.617 (0.560–0.673)	26.8 (17.0–39.6)	86.2 (82.1–89.6)	25.0 (15.8–37.2)	87.3 (83.2–90.5)	1.95 (1.17–3.24)	0.85 (0.72–1.00)
Bova	0.663 (0.587–0.740)	14.3 (7.4–25.7)	95.7 (92.9–97.4)	36.4 (19.7–57.0)	86.7 (82.8–89.8)	3.34 (1.47–7.58)	0.90 (0.80–1.00)
modified FAST	0.680 (0.608–0.752)	53.6 (40.7–66.0)	74.0 (69.0–78.5)	26.1 (18.9–34.8)	90.3 (86.2–93.3)	2.06 (1.52–2.80)	0.63 (0.47–0.84)

AUC: Area under curve, CI: Confidence interval, LR: Likelihood ratio. NPV: Negative predictive value, PPV: Positive predictive value. AUC with DeLong 95% CI; sensitivity/specificity/PPV/NPV with Wilson 95% CI, at each model’s intermediate–high-risk threshold.

**Table 5:** Pairwise AUC comparisons and classification agreement.

Comparison	ΔAUC	DeLong *p* (Raw)	DeLong *p* (Holm)	Cohen’s κ (95% CI)
ESC vs. Bova	−0.046	0.200	0.401	0.414 (0.279–0.549)
ESC vs. mFAST	−0.063	0.110	0.329	0.115 (0.016–0.214)
Bova vs. mFAST	−0.017	0.693	0.693	0.216 (0.130–0.303)

AUC: Area under curve. ΔAUC and DeLong *p* (raw and Holm-adjusted across 3 comparisons); Cohen’s κ with 95% CI between binary intermediate–high-risk flags.

## Data Availability

The dataset is available from the corresponding author on reasonable request; patient-level data are held locally in accordance with the ethics approval.

## Abbreviations

The following abbreviations are used in this manuscript:

AUC	Area under the curve
CAD	Coronary artery disease
CI	Confidence interval
CKD	Chronic kidney disease
CTPA	Computed tomography pulmonary angiography
DVT	Deep vein thrombosis
ESC	European Society of Cardiology
ICU	Intensive care unit
IQR	Interquartile range
LV	Left ventricle
RV	Right ventricle
SBP	Systolic blood pressure
sPESI	Simplified Pulmonary Embolism Severity Index
SpO_2_	Peripheral oxygen saturation
